# Disentangling Population Health Management Initiatives in Diabetes Care: A Scoping Review

**DOI:** 10.5334/ijic.7512

**Published:** 2024-01-30

**Authors:** Rose J. Geurten, Jeroen N. Struijs, Henk J. G. Bilo, Dirk Ruwaard, Arianne M. J. Elissen

**Affiliations:** 1 Department of Health Services Research, CAPHRI Care and Public Health Research Institute, Faculty of Health, Medicine and Life Sciences, Maastricht University, Maastricht, P.O. Box 616, 6200 MD Maastricht, The Netherlands; 2 Department of Quality of Care and Health Economics, Center for Nutrition, Prevention and Health Services, National Institute of Public Health and the Environment (RIVM), Bilthoven, The Netherlands; 3 Leiden University Medical Centre, Department Public Health and Primary Care - Campus The Hague, The Hague, P.O. Box 1, 3720 BA Bilthoven, The Netherlands; 4 Department of Internal Medicine, University of Groningen and University Medical Center Groningen, Groningen, Diabetes Research Center, Mondriaangebouw, Dokter van Deenweg 1-10, 8025BP Zwolle, the Netherlands

**Keywords:** population health management, population management, diabetes, type 2 diabetes

## Abstract

**Introduction:**

Population Health Management (PHM) focusses on keeping the whole population as healthy as possible. As such, it could be a promising approach for long-term health improvement in type 2 diabetes. This scoping review aimed to examine the extent to which and how PHM is used in the care for people with type 2 diabetes.

**Methods:**

PubMed, Web of Science, and Embase were searched between January 2000 and September 2021 for papers on self-reported PHM initiatives for type 2 diabetes. Eligible initiatives were described using the analytical framework for PHM.

**Results:**

In total, 25 studies regarding 18 PHM initiatives for type 2 diabetes populations were included. There is considerable variation in whether and how the PHM steps are operationalized in existing PHM initiatives. Population identification, impact evaluation, and quality improvement processes were generally part of the PHM initiatives. Triple Aim assessment and risk stratification actions were scarce or explained in little detail. Moreover, cross-sector integration is key in PHM but scarce in practice.

**Conclusion:**

Operationalization of PHM in practice is limited compared to the PHM steps described in the analytical framework. Extended risk stratification and integration efforts would contribute to whole-person care and further health improvements within the population.

## Introduction

The increasing burden of chronic disease leads to concerns regarding the financial sustainability of health systems [1,2]. Already, many countries face challenges in providing accessible and affordable high-quality care [2,3]. Type 2 diabetes is a highly prevalent chronic disease and its prevalence is growing on a global scale [4]. Worldwide, an estimated 463 million patients (9.3% of the adult population in 2019) have type 2 diabetes [4,5]. In the Netherlands alone, type 2 diabetes affects 1 million people (in 2018) [6]. Persons with diabetes often have high needs and high costs of care: of the top 10% of care utilizers, 39% has diabetes (compared to 9% of all adults) [4,7]. Prior research has shown that persons with type 2 diabetes tend to use care across multiple healthcare sectors and from various medical specialties [6,8]. Much of their service use is related to treating emergent problems such as comorbidities and/or diabetes-related complications, which are known to increase care use and expenditures [8,9,10,11]. This implies that while care for type 2 diabetes in itself is not necessarily expensive, whole-person care for patients with type 2 diabetes is costly [8,11]. The diverse health risks, care use and care expenditures of type 2 diabetes populations are in part caused by heterogeneity in socio-demographic backgrounds [6,8,12,13]. Factors such as ethnicity, income, and level of education are associated with differences in diabetes risk as well as care utilization and outcomes [14,15]. For example, low levels of education and low health literacy increase type 2 diabetes prevalence and negatively affect the ability to manage the disease [14,15,16,17]. Furthermore, patient characteristics such as age, disease duration and Body Mass Index (BMI) influence glycaemic control in type 2 diabetes patients [12,18]. Accordingly, current standardized and health care oriented diabetes care approaches seem too limited to address the heterogeneous population needs [9].

It is increasingly evident that to improve the health of large, heterogeneous populations, such as the type 2 diabetes population, the focus should be broader than strictly medical care. New initiatives need to bridge gaps and aim to integrate services across health care, health promotion and prevention, social care and welfare [19]. Approaches that aim to bridge these gaps are often labelled as Population Health Management (PHM) [20]. Moreover, PHM initiatives strive to address health needs at all points along the continuum of health for a specified population and include both health outcomes and determinants of health [20,21,22]. By doing so, the goal is to organize a proactive health system around a population and to improve their care and reduce health care costs by keeping the whole population as healthy as possible [17,23,24]. To achieve this, PHM includes data-driven population identification and risk stratification methods to develop personalized interventions that target the identified subpopulations with different health status and health risks [20,25]. As such, PHM can contribute to answering questions regarding what works, for whom, and in what context. PHM may therefore be a promising approach to long-term health improvement and prevention of type 2 diabetes and related complications; this may subsequently lead to a stabilization and possibly a reduction of overall costs, promoting the sustainability of healthcare systems [8,9,11,17,26].

Although PHM for people with type 2 diabetes, other prevalent chronic diseases, or heterogeneous populations seems promising, to date, insight into initiatives that integrate and tailor services across health promotion and prevention, health care, and social services is limited. Therefore, this scoping review explores the state of knowledge and developments in PHM initiatives for people with type 2 diabetes. The aim of this review was to gain insight into the extent to which and how PHM is used in whole-person care for people with type 2 diabetes. The insight into the status quo can contribute to shaping future research directions.

## Methods

### Study design

A scoping review was performed to map relevant peer-reviewed literature and identify the current state of practice in the field of PHM for people with type 2 diabetes [27,28]. Scoping reviews are suitable to address such broad research questions and/or topics, and in turn can serve to develop more specific research questions to address identified gaps. Unlike a systematic review, scoping reviews do not provide a formal quality evaluation of included papers and generally do not aim to evaluate outcomes [27,28]. This review used the 5-step scoping review method [27] and the extended PRISMA (Preferred Reporting Items for Systematic Reviews and Meta-Analyses) framework for scoping reviews (PRISMA-ScR) [28,29]. This includes: (1) identifying the research question; (2) identifying relevant studies; (3) selecting studies; (4) charting the data; (5) collating, summarizing and reporting results.

### Identifying relevant studies

The literature search was conducted in PubMed, Web of Science, and Embase and was limited to English papers published between January 2000 and September 2021. The search strategy was built on the 2017 scoping review of Steenkamer et al. [30] regarding the definition of PHM, this review identified papers that mention “population health management” (PHM) or “population management” (PM) in the title and/or abstract to map relevant literature. For the aim of this study, we searched for papers that additionally mention “diabetes” in title and/or abstract. MeSH terms or Emtree terms were used for PHM in Pubmed and Web of Science, respectively. The search strings are shown in Appendix Table 1. After identifying the eligible papers, additional studies about the included PHM initiatives were identified using the snowball method. This was done in mid-January 2022, all related papers up to that point were included.

### Selecting studies

First, title and abstract were reviewed for study eligibility. Eligible papers explicitly described a self-reported PHM or PM initiative for type 2 diabetes and full-text was available. An initiative for type 2 diabetes additionally included initiatives for both type 2 and another type of diabetes (e.g. type 1), and initiatives for type 2 diabetes patients with a specific complication, comorbidity, or additional condition. These eligibility criteria are shown in Appendix Table 2.

Two authors (RG and AE) independently reviewed title and abstract of 25% of identified studies to determine if these met all eligibility criteria. Discrepancies and uncertainties about study inclusion were discussed. As there were no disagreements, RG reviewed the remaining 75% of studies. The web app of Rayyan [31] was used for title and abstract screening. Full-text screening and data extraction were done using Mendeley reference manager.

### Charting the data

Charting tables to extract key information from the eligible articles included : (1) the source (i.e. author, reference); (2) PHM initiative type and details (i.e. origin, description of initiative, target population, setting); and (3) details on PHM actions in each initiative to examine the extent to which and how PHM is used in whole-person care for people with type 2 diabetes [28,29]. For the latter, each initiative was disentangled based on Struijs et al.’s (2014) [20] analytical framework for PHM. This analytical framework (adapted from The Care Continuum Alliance [CCA] model) was developed to establish, evaluate, and compare PHM initiatives, and comprises six subsequent steps:

1. Population identification. The identification of the target population based on specific characteristics or criteria;
2. Triple Aim assessment. As the aim of PHM is to achieve improvements on the Triple Aim domains (i.e. population health, quality of care, direct and indirect costs), a detailed ‘snapshot’ of these domains within the identified population is needed to provide input on interventions needed;
3. Risk stratification. Stratification of the identified individuals into subpopulations, for instance, based on outcomes of step 2. These subpopulations are meaningful to tailor interventions and efficiently allocate resources;
4. People centred interventions. Subdivided into two types of interventions: a.) tailored interventions for predefined subpopulations (e.g. determined in step 3) and b.) interventions aimed to realize or improve the prerequisites for PM (closely related to and part of the contextual factors, e.g. staff training or alignment of incentives between providers);
5. Impact evaluation. Evaluation of PHM initiatives based on indicators measuring the overarching goals of PM, i.e. the Triple Aim and related domains. For a complete insight, evaluation of the domains as defined by Hendrikx et al. (2016) [23] (e.g. health outcomes, behavioural and psychological factors, and responsiveness) was assessed; and
6. Quality improvement processes. Continuous improvement cycles (e.g. Plan-Do-Study-Act [PDSA] or Plan-Do-Check-Act [PDCA] cycles).

To examine the extent to which and how PHM is used in whole-person care for people with type 2 diabetes, whether and how each of the six PHM steps were undertaken in the PHM initiatives was described. In addition, a data warehouse that integrates Triple Aim information is essential in PHM initiatives [25]. Specifically, data use is necessary for the PHM steps of population identification, Triple Aim assessment, risk stratification, and impact evaluation [19,20,25]. Therefore, the availability and use of a data warehouse within each of the PHM initiatives for type 2 diabetes was assessed.

## Results

**Figure 1:**
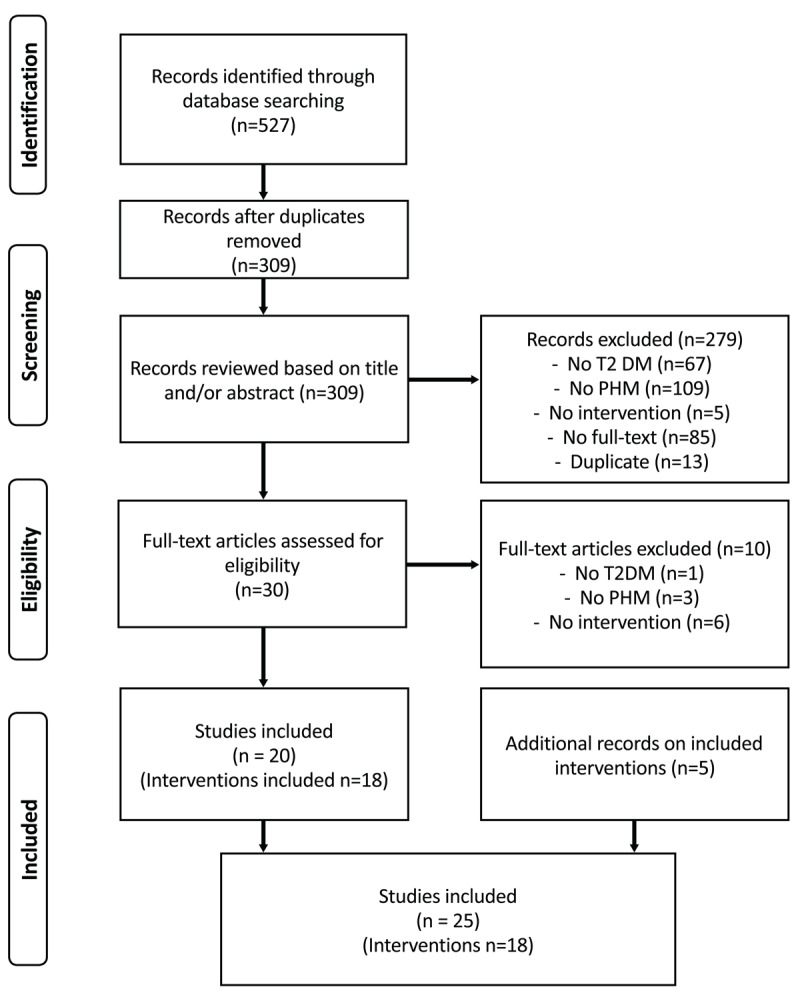
Scoping review flow chart.

The PRISMA diagram in Figure 1 shows the literature search and screening process. In total 527 records were identified through database searching, 208 duplicates were removed. Of the 309 distinct articles found in the literature search, 30 were considered relevant based on title/abstract. These articles were retrieved and full-text was assessed for eligibility. Of the 30 full articles reviewed, 20 articles met the eligibility criteria. Through snowballing, five additional studies related to three eligible initiatives were included: three studies related to the Endo ECHO project [32,33,34], one to the INDEPENDENT initiative [35,36], and one to the DM-PEP initiative [37]. In total, 25 studies that described 18 unique self-reported PHM initiatives for type 2 diabetes populations were included. The number of eligible studies published per year increased over the study period, with no publications before 2003 and seven publications in 2020 alone (Appendix Figure 1).

### PHM initiatives

The 18 unique self-reported PHM initiatives for type 2 diabetes populations are described in Appendix Table 3. The majority originated from the United States (US) (15/18) [16,32,43,44,45,46,47,48,49,50,51,52,33,53,34,37,38,39,40,41,42]. Two initiatives were implemented in the United Kingdom (UK) [54,55] and one in India [35,56]. The initiatives were most often described as ‘population management’ (PM; n = 11) [16,41,53,54,42,43,44,46,47,48,50,51], followed by ‘population health management’ (PHM; n = 7) [37,38,39,40,45,49,52,55,56]. The majority of initiatives were situated in the primary care sector (12/18) [16,38,49,51,52,54,39,40,42,43,44,45,46,47]. Three initiatives were in hospital care [35,50,53,56] and one initiative in pharmaceutical care [48]. The PHASE program was initially implemented in hospital care [50] and subsequently expanded to multiple settings: hospital, regional clinics, and community health centres [37]. The remaining two initiatives were implemented across multiple settings. Project Endo ECHO focused on the continuity of care across community health centres and an academic medical centre by telementoring appointments [32,33,34,41]. The integrated diabetes care pilot aimed to create collaborative working between primary, secondary, and community care [55]. The majority of initiatives (12/18) were developed to be used by providers to, in turn, improve care for diabetes patients (e.g. registries, medical record review) [16,32,46,49,52,53,55,56,33,34,35,38,41,43,44,45].

### PHM steps

This section describes whether, and to what extent, the six steps of the analytical framework for PHM were performed in the self-reported PHM initiatives. Appendix Table 4 shows how population identification was performed, whether and how risk stratification was done, and gives insight into actions related to people centred interventions. Table 1 shows what steps were undertaken related to the Triple Aim assessment prior to the initiatives (Appendix Table 5 describes these in detail). Table 2 displays which domains of the Triple Aim were assessed for impact evaluation of the initiatives. Quality improvement processes performed are explained (Appendix Table 6) and, lastly, the availability and use of a data warehouse within the PHM initiatives is described (Appendix Table 7).

**Table 1:** Triple Aim assessment (step 2) before implementation of PHM interventions for type 2 diabetes.


PHM INITIATIVES WITH TRIPLE AIM ASSESSMENT	ASSESSMENT ACTIONS

**PROPS Study (Partnerships for Reducing Overweight and Obesity with Patient-centred Strategies) [PHM]** [39,40]	**Quality of care**: eligible patients were asked about their motivation to lose weight and only patients who were motivated were eligible (**responsiveness**)

**Project Endo ECHO: Telementoring for care providers [PM]** [32,33,34,41]	**Quality of care**: prior to the launch, the primary care clinicians and community health workers participated in a 2-day face-to-face training (**safety**)

**Integrated disease management [PM]** [46]	**Quality of care**: dissemination of program in three phases, each building on prior experiences (initial design, formal pilot, broad program dissemination)

**INtegrating DEPrEssioN and Diabetes treatmENT (INDEPENDENT) care [PHM]** [35,56]	**Quality of care**: potential participants were approached whether they were interested to participate **(responsiveness)**, cultural modifications were made based on formative research **(responsiveness)**, prior to patient enrolment teams at each site received training in process measures and treatment measures within the initiative **(safety)**

**Integrated diabetes care pilot [PHM]** [55]	**Quality of care:** clinical audit to help target resources

**Mobile health (mHealth) Self-Management Intervention [PHM]** [47]	**Quality of care**: all intervention components were designed to be culturally sensitive, and content addressed self-management facilitators and barriers common in this population (**responsiveness**), eligible patients could opt out of the study (**responsiveness**)

**Population-based evidence-based medicine [PM]** [48]	**Quality of care**: patients had the option to decline starting or increasing lisinopril therapy (**responsiveness**)

**Preventing Heart Attacks and Strokes Everyday (PHASE) program [PHM]** [37,50]	**Quality of care**: specially trained diabetes care manager nurses and pharmacists support the primary care team (**safety**)

**The Diabetes Master Clinician Program (DMCP) [PM]** [51]	**Quality of care**: training for clinicians, medical assistants, nurses (**safety**)

**International community health service PHM program (multilingual) [PHM]** [52]	**Quality of care**: PHM interventions could be offered, received, or refused (**responsiveness**)

**Registry population management [PM]** [53]	**Quality of care**: pre-existing problems related to **effectiveness** (unclear effectiveness of interventions, inefficient tracking and managing patient data, and poor information exchange) and **accessibility** (no tracking and fewer interventions for non–pay-for-performance patients) were addressed


**Table 2:** Domains of the Triple Aim assessed for impact evaluation in PHM initiatives for type 2 diabetes.


**Population health**	Health outcomes	•	•	•	•	•	•	•	•	•		•	•	•	•	•	•		•

	Disease burden																		

	Behavioural/psychological factors		•	•						•									

	Participation					•						•							

	Functioning/QoL		•							•									

**Quality of care**	Quality of care	•		•			•	•	•		•						•		

	Patient safety												•						

	Effectivity		•	•							•			•				•	

	Responsiveness		•	•	•		•			•						•			

	Timeliness																		

	Support	•						•							•				

	Accessibility			•					•										

**Costs**	Costs of care			•						•				•		•			

	Volume																		

	Costs PM organization			•														•	

	Productivity losses (indirect)									•									

**PHM initiatives refs:**		[38]	[39,40]	[32,33,34,41]	[54]	[42]	[43,44]	[45]	[46]	[35,56]	[55]	[47]	[48]	[49]	[37,50]	[51]	[52]	[53]	[16]


*Notes*: • indicates that the specific domain was evaluated in the initiative. Empty cells indicate that the specific domain was not evaluated in the initiative. Grey areas indicate that no domains of that Triple Aim dimension were evaluated in the initiative.

#### Population identification

The first PHM action ‘population identification’ was done in all PHM initiatives (Appendix Table 4). Nine initiatives focused on all patients with diabetes [37,42,54,55,43,44,45,46,49,50,51,53]. Three initiatives specifically focused on patients with high-risk or complex diabetes [16,32,33,34,41,43]. This was determined based on various criteria, which always involved glycated haemoglobin (HbA1c) levels. Two of the 18 initiatives were for type 2 diabetes alone [39,40,47]. Five initiatives targeted patients with diabetes and an additional criterion or condition, being: BMI between 27 and 40 kg/m2 [39,40], depressive symptoms and a poorly-controlled cardio metabolic indicator [35,56], coronary artery disease (CAD) [48], or hypertension [47,52]. One initiative was designed to target patients with diabetes or patients with other conditions (cardiovascular disease (CVD) or hypertension (HTN)) [38].

#### Triple Aim assessment

The Triple Aim is not widely used to asses population needs prior to implementation of the identified PHM initiatives (Table 1). None of the PHM initiatives assessed population health and direct or indirect costs before implementation. In total, 11 of 18 PHM initiatives assessed quality of care before the start of the initiative [32,33,48,50,51,52,53,56,34,35,37,39,40,41,46,47]. This was done in multiple ways. Responsiveness was ensured by cultural modifications prior to implementation [35,47,56] and by the option for participants to decline participation or opt out [35,39,40,47,48,52,56]. Safety was assured by staff training prior to the initiative [32,33,34,35,37,41,50,51,56]. One initiative assessed quality of care by stepped dissemination of the program, each building on prior experience [46]. Another initiative addressed known pre-existing problems relating to effectiveness and accessibility [53]. Lastly, a pilot study used clinical audits to help target resources [55]. In addition to quality of care, provider experience was weighed prior to three initiatives by enabling customization at implementation [46] and using feedback on feasibility and acceptability as input for the final care model [35,55,56]. The population need for the initiatives is generally reasoned based on literature.

#### Risk stratification

The majority of initiatives included risk stratification (12/18) where only three of these initiatives explicitly mention risk stratification [46,54,55]. In general, PHM initiatives that initially focus on all patients with diabetes apply risk stratification (n = 8) [37,42,45,46,50,51,53,54,55] and PHM initiatives focusing on a specific segmentation (e.g. high-risk diabetes) do not further stratify the identified population (n = 6) [32,33,57,34,38,39,40,41,43,44,52]. Three initiatives both selected a specific segmentation and applied risk stratification [16,35,48,56]. One initiative focused on diabetes patients with no risk stratification (Appendix Table 4) [49].

Risk stratification was generally based on clinical measures, most common were: HbA1c [35,42,48,51,56], blood pressure (BP) [35,42,48,51,54,56], and low-density lipoprotein (LDL) [35,42,51,56]. In two initiatives, the risk stratification was determined by the healthcare provider rather than based on specified clinical prerequisites [16,45]. One initiative integrated three major evidence-based national treatment guidelines to make a clinical risk-stratification algorithm, however, prerequisites used were not clarified [46]. In addition, three initiatives used registry software that stratified the population [35,53,56]. For two of these initiatives, the prerequisites remain unclear as these only mention that the dashboard and application help “prioritizing participants for follow-up” [56] and “determine cohorts of patients with specific criteria and needs” [53]. In general, risk stratification prerequisites are not explained in detail. Some initiatives have little detail and do not specify any factors used to determine risk [16,45,50]. The majority of initiatives has medium detail and show the factors on which stratification is based but do not disclose specific measures related to various risk levels [35,42,46,51,53,54,56]. For two initiatives, risk stratification methods were described in such detail that it enables applying these methods to other diabetes population [48,55].

#### People centred interventions

##### Tailored interventions for predefined subpopulations

Care was tailored in 13 of the PHM initiatives. In five initiatives, care was not described [45] or not tailored to individual or subpopulation needs (Appendix Table 4) [32,33,34,38,39,40,41,46]. The tailored interventions mostly targeted patients on the individual-level (n = 9) [16,35,56,42,43,44,47,49,50,52,54], specific cohorts (e.g. high-risk patients) were targeted in a lesser extent (n = 4) [48,51,53,55]. In line, initiatives were most commonly tailored using individualized recommendations, goals, interventions, or educational classes [16,35,43,44,47,49,50,56]. Other methods described to tailor care were prioritization of (telephone) contacts or support [35,42,56], adaptation of the monitoring interval [54], medication schedules [48], group visits [51], and language [52].

##### Interventions aimed to realize or improve the prerequisites for PM

Actions to realize or improve prerequisites were found in all but two initiatives [42,48]. The most common action was registry development, specifically: electronic health record (EHR) tool or system development [35,38,47,56]; diabetes registry development [45,51,54]; and clinical software, review program, or application development [43,44,49,53]. A second common method to realize or improve the prerequisites for PM was through staff: training staff [32,33,34,35,37,41,51,52,56], having specific PHM staff or diabetes care staff [16,37,39,40,50], or assigning a population health coordinator [38,56]. System redesign and/or integration of care [16,35,37,46,52,55,56] as well as collaborative care [35,55,56] are additional methods used to achieve this goal. In one initiative, a change agent was appointed to ensure readiness for change [45].

#### Impact evaluation

Sixteen interventions looked at population health for impact evaluation, all of these assessed health outcomes [16,33,43,44,45,46,47,48,49,50,51,52,34,54,56,35,37,38,39,40,41,42]. Quality of care was used to assess impact in 15/18 interventions [32,33,44,45,46,48,49,50,51,52,53,54,34,55,56,35,37,38,39,40,41,43]. Impact was hardly evaluated based on costs, which were never discussed or explained in detail (5/18) [34,35,41,49,51,53,56]. The assessed domains per Triple Aim dimension are displayed in Table 2, in Appendix Table 5 these actions are described in detail.

##### Population health

The assessment of population health is generally based on health outcomes (Table 2) as 16/18 initiatives assessed health outcomes. This was done based on clinical measures (e.g. HbA1C, BP, LDL) [16,35,49,50,51,52,54,56,37,38,39,40,41,42,45,46]; metabolic risk factors [43,44]; clinical testing [33]; or medication, comorbidities, healthcare use, and further medical history [34,44,45,46,49]. Health was additionally assessed based on behavioural/psychological factors (3/18) [32,33,34,35,39,40,41,56], functioning/QoL (2/18) [35,39,40,56], and participation (2/18) [42,47]. Two initiatives did not evaluate impact on population health [53,55].

##### Quality of care

Impact on quality of care was assessed in 15/18 initiatives (Table 2) [33,34,55,56,35,37,38,44,45,46,50,52]. This was done by assessing changes in frequency of clinical goal attainment [34,38,52], clinical testing [33,44] and/or use of guideline-based therapy [32,33,34,35,41,52,56]; and evaluating changes in organizational or staffs’ performance or knowledge [45,46,49,55]. Responsiveness was assessed in six initiatives, this was generally done by taking patient input, feedback or satisfaction into account [35,39,40,41,51,54,56]. One initiative assessed the percentage of specific recommendations on clinical testing and changes in therapy [43,44]. Three initiatives used a registry to support quality of care as these were used to evaluate process and outcome measures [37,38,45,50]. Other evaluations related to changes in effectivity [32,33,34,39,40,41,49,53,55] and accessibility [32,33,34,41,46]. Two initiatives did not address quality of care.

##### Direct and indirect costs

The minority of studies assessed costs: five initiatives assessed direct and indirect [41,56] or direct costs only [34,35,49,51,53]. Moreover, when costs are assessed this is not discussed or explained in detail. The remainder of initiatives did not mention cost assessment [16,32,45,46,47,48,50,52,54,55,33,37,38,39,40,42,43,44].

#### Quality improvement process

Quality improvement processes were in place in 10 of 18 initiatives, four used a PDSA or PDCA cycle (Appendix Table 6) [38,45,46,53]. The remaining initiatives implemented quality improvement by: stakeholder and user input/feedback [38,39,52], phased program development [46,49], clinical audits and learning culture [55], tracking data [48], regular reports for a quality improvement program and individual quality improvement coaching [37,50]. Nine initiatives did not have quality improvement processes [16,32,47,48,51,54,56,33,34,35,40,41,42,43,44].

#### Data warehouse availability and use

A data warehouse was available in 11/18 of the PHM initiatives [35,37,49,50,52,53,56,38,39,40,42,43,44,47,48]. Appendix Table 7 shows the type of data available and for which PHM step(s) the data was used. The available data were generally clinical: data from a clinical (diabetes) registry [37,38,42,50], electronic health records (EHR) [39,40,47,52], or (electronic) medical records [35,43,44,56]. Three PHM initiatives combined multiple data sources. One PHM initiative used a database combining membership, administrative, and clinical data [48]. Another initiative used a combination of medical records, payer data, and a registry of diabetes patients [49]. Lastly, a registry population management application aimed to improve the workflow by combining clinical, claims, laboratory, and administrative data [53]. In all initiatives with a data warehouse available, the data were exclusively used for population identification.

## Discussion

This scoping review was performed to explore the extent to which and how PHM is used in whole-person care for people with type 2 diabetes. PHM is a promising approach to long-term health improvement in chronic disease populations, which may lead to a stabilization and possibly a reduction of overall costs, promoting the sustainability of healthcare systems [8,9,11,17,26]. This scoping review shows variation in whether, to what extent, and in what way, the six steps of the analytical framework of PHM were performed in the PHM initiatives for people with type 2 diabetes mellitus. Population identification, interventions to realize or improve prerequisites for PM (part of people centred interventions), impact evaluation, and quality improvement processes were generally part of the PHM initiatives. Risk stratification and tailored interventions (part of people centred interventions) were described in the majority of initiatives but details on these rather relevant points were limited. Triple Aim assessment prior to the initiative was scarce. Moreover, there was no PHM initiative that used a data warehouse to address the Triple Aim to inform interventions needed. Additionally, there are differences in to what extent and how the PHM steps are realized. For example, in impact evaluation, population health was assessed in almost all initiatives whereas costs and provider experience was assessed in the minority of initiatives. As for content, population identification focused on the whole diabetes population as well as specific subgroups, and care was tailored to individual as well as subpopulation needs.

The variation in whether and how the six PHM steps were performed in our included studies in type 2 diabetes care may be due to the ongoing differences in conceptualizations of PHM [20,30]. A scoping review of Steenkamer et al. (2017) [30], performed to define PHM, found that not only the definition but also the performance of the six PHM steps show variation. PHM definitions range from goalsetting for a specific initiative to descriptions of what PHM entails. For the latter, the minority of PHM definitions relate to earlier descriptions of PHM, meaning new PHM definitions are continually created [30]. An additional point of variation in PHM regards integration of care. One regularly described strength of PHM is that it can bridge gaps and integrate services across health care, health promotion and prevention, social care and welfare [19,20]. As such, PHM enables proactive, tailored, whole-person care around a population to improve their care and prevent type 2 diabetes and related complications [17,20,23,24]. However, we found the majority of PHM initiatives for type 2 diabetes were situated within one health care setting (16/18), and just two initiatives implemented across different settings. This was the case in a study involving health centres and academic medical centre [32,33,34,41], and a study assessing the effects of collaborative working between primary and secondary care in the community setting [55]. These integrated services aim to improve diabetes care and outcomes by integration but are still limited to the healthcare sector. Given the influence of non-clinical factors (i.e. socio-demographic background and patient characteristics) on their health, integrating services across sectors is particularly relevant for type 2 diabetes patients [6,8,12,13,18]. However, collaborating across sectors is difficult and the complexity of integration tends to grow as the distance between organizations expands. Previous research points to challenges in sharing data (practical and legal), joint financial management, and social features of integration (e.g. differences in culture and alignment of incentives) [19,58,59,60]. These challenges could be overcome by power-sharing, building a collaborative culture, clear roles and responsibilities, and effective communication strategies between sectors [61]. Despite the complexity of cross-sector integration, the discrepancy between the regularly described integration across sectors in PHM compared to what happens in practice further emphasizes the difference in interpretation of PHM. Moreover, the lack of integration across sectors limits PHM’s potential to provide whole-person, integrated care whereas that broad view could contribute to prevent type 2 diabetes and related complications and subsequently promote the sustainability of healthcare systems [8,9,11,17,26].

Another discrepancy in the operationalization of PHM lies in risk stratification. Two types of risk stratification within the PHM initiatives can be recognised: initiatives that initially focus on all patients with diabetes and apply risk stratification [37,42,45,46,50,51,53,54,55], and initiatives focusing on a specific segment of the diabetes population (e.g. high-risk patients) which do not stratify the identified population [32,33,57,34,38,39,40,41,43,44,52]. Thus, the former seems to tailor interventions whereas the latter targets the intervention. Interventions targeted at high-risk patients are limited in comparison to PHM which generally strives to organize proactive health care around a population, to address health needs at all points along the continuum of health, and to improve care and reduce health care costs by keeping the whole population as healthy as possible [17,20,21,23,24]. Moreover, risk stratification of patients was often limited to clinical measures, rather than based on a whole-person paradigm of health. This narrow view may negatively influence the potential of PHM initiatives to address what works, for whom, and in what context, and ultimately hampers initiatives to prevent type 2 diabetes and related complications. Amongst others, this is since variations in health care use is largely related to heterogeneity in socio-demographic factors [6,12,13]. Socio-demographic factors such as ethnicity, income, and level of education influence diabetes prevalence as well as care outcomes [14]. Additionally, patient characteristics such as age, disease duration, and BMI influence glycaemic control in type 2 diabetes patients [12,18]. However, prior risk stratification efforts are generally based on clinical factors as well. Risk stratification for type 2 diabetes is often solely based on clinical measures such as HbA1c, blood pressure, presence of diabetes-related complications, and insulin use [62,63,64]. Only few stratification methods use non-traditional variables such as health behaviours and beliefs [65].

The narrow view in stratification variables may partly be due to limits in available data. Large and coupled datasets including data on sociodemographic factors as well as the necessary clinical data are seldom available. In PHM, a data warehouse is essential to enable population identification, Triple Aim assessment, risk stratification, impact evaluation and in turn, whole-person care [19,20]. However, in PHM initiatives the availability of data is similarly limited. Data warehouses were used in 11/18 of the PHM initiatives for type 2 diabetes, however; only three sources included other data in addition to clinical data. These three data warehouses were still not detailed enough to support whole-person care as these additions remained limited to membership, administrative, and payer or claims data [48,49,53]. Moreover, existing data sources were only used to identify the population and not to inform on the status quo of the population on the Triple Aim dimensions, risk stratification, and impact evaluation. Prior research similarly showed limited use of data in PHM initiatives [23,30]. This limited use of coupled data may be caused by practical (e.g. different IT systems) and legal barriers [58,59]. Future IT developments should ideally integrate all relevant data regarding health (i.e. clinical, socio-demographic, and other non-medical determinants of health) [6,8,12,13]. In recent years, such regional cross-sector partnerships have emerged in several countries. An example close to home is the Dutch regional integrative population-based data infrastructure Extramural LUMC (Leiden University Medical Centre) Academic Network (ELAN) which links routinely collected medical, social, and public health data at the patient level [66]. They found that to overcome practical and legal barriers, prerequisites to develop such a data structure are executive-level support, overcoming privacy and legislation concerns together, taking time, and establishing reciprocity in data sharing [66]. Future initiatives may benefit from implementing these conditions when combining data.

To our knowledge, this is the first scoping review to explore the extent to which and how PHM for people with type 2 diabetes is used in practice. Despite the lack of a clear definition of PHM [20,30], we were able to compare activities, contextual factors, and operationalization of PHM initiatives by disentangling the initiatives based on an analytical framework [20]. Our findings have implications for all PHM initiatives that aim to provide whole-person care to heterogeneous populations and, thus, may be meaningful for PHM policymakers, professionals and researchers. Our search was limited to self-reported PHM initiatives for people with type 2 diabetes, i.e. initiatives that mentioned “population health management” or “population management” in the title and/or abstract. Thus, we may miss PHM initiatives, which pursue population management but do not explicitly mention PHM and/or PM. However, including such papers would leave room for discussion as a clear definition of PHM is lacking [20,30]. As this is a scoping review, we did not analyse the outcomes of initiatives. However, this seems appropriate for our aim to assess the extent to which and how PHM is used in whole-person care for people with type 2 diabetes. With this insight, we consider it meaningful to assess opportunities to improve cross-sector integration as well as development of coupled data warehouses in PHM initiatives in future research. These topics are crucial to achieve pro-active, whole-person, and people centred interventions, which are key benefits of PHM.

## Conclusion

Disentangling and analysing the components of the reported PHM initiatives reveals variation in operationalization of PHM. Where population identification, improvement of PHM prerequisites, impact evaluation, and quality improvement processes were generally part of the PHM initiatives, integration over sectors, Triple Aim assessment, and risk stratification actions were scarce or explained in little detail. This additionally shows a discrepancy in operationalization of PHM in practice compared to theory: the self-reported PHM initiatives often have a narrower and more targeted approach than would be expected based on the PHM framework. This limits PHM’s potential to provide whole-person, integrated care and achieve the intended outcomes. Extending risk stratification and integration efforts could, therefore, contribute to whole-person care and further health improvements within the population. Despite the variation in whether, to what extent, and how the six steps of PHM are performed, PHM for people with type 2 diabetes is increasingly used and seems promising. However, the variation in PHM asks for further clarification of how to operationalize PHM.

## Additional File

The additional file for this article can be found as follows:

10.5334/ijic.7512.s1 Appendix.
