# Supplementary material for: Disentangling Population Health Management Initiatives in Diabetes Care: A Scoping Review
Source: Int J Integr Care. 2024 Jan 30;24(1):3. doi: 10.5334/ijic.7512 (PMC10836183; doi:10.5334/ijic.7512)
Supplement: Appendix. [file ijic-24-1-7512-s1.pdf]

**Appendix Table 1 Search strategies**
**Pubmed**
(((population health management[MeSH Terms]) OR (population health management[Title/Abstract])) OR (population management[Title/Abstract])) AND (diabetes[Title/Abstract])<br><br>Filters: from 2000/1/1 – 2021/9/30
**Web of Science**
(((TI=("population health management") OR AB=("population health management")) OR (TI=("population management") OR AB=("population management"))) AND (TI=(diabetes) OR AB=(diabetes))<br><br>Timespan: 2000-01-01 to 2021-09-30 (Index Date)
**Embase**
1. “population health management”.ab,ti.<br>2. exp population health management/<br>3. ““population management”.ab,ti.<br>4. Diabetes.ab,ti.<br>5. 1 or 2 or 3<br>6. 4 and 5

Appendix Table 2 Eligibility criteria		
	**Inclusion**	**Exclusion**
**Population**	People with type 2 diabetes, or;<br>People with type 2 diabetes and people with another type of diabetes, or;<br>People with type 2 diabetes and a specific complication, comorbidity, or additional condition	Only people with another type of diabetes (not type 2)
**Initiative**	Self-reported PHM or PM	Initiatives that implement key aims of PHM/PM but do not self-report to be PHM/PM
**Language**	English	All other languages
**Study design**	All	-
**Type of publication**	Published in peer-reviewed journals<br>Full-text available	Grey literature<br>No full-text available
**Date of publishing**	Between January 2000 and September 2021	Before January 2000 or after September 2021

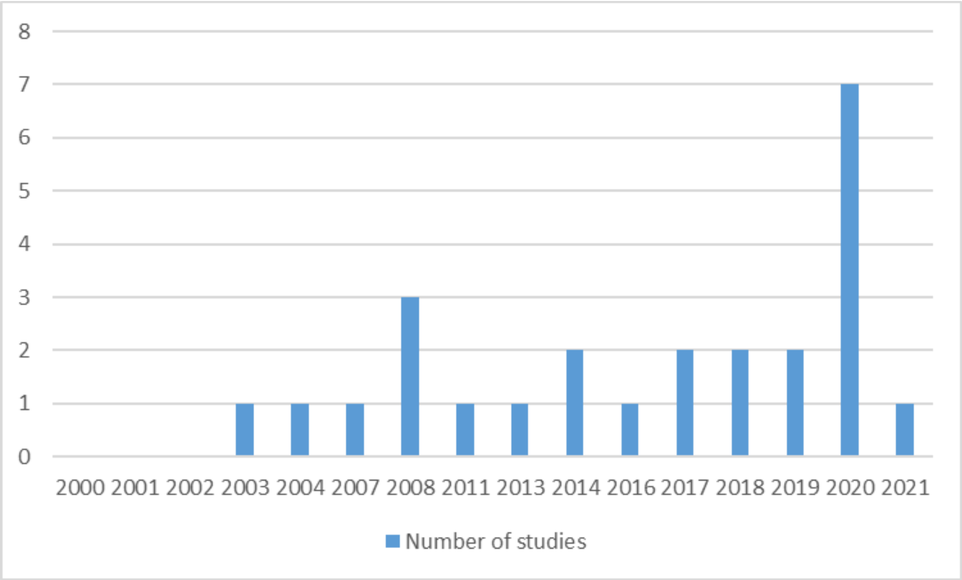

Appendix Figure 1 Publication years of eligible studies on PHM for diabetes

**Appendix Table 3 PHM initiatives setting and description**		
**Initiative and origin**	**Setting**	**Description**
**Health information technology–enabled PHM program [PHM]**[1]	Primary care	Health information technology (clinical registry) enabled Population Health Management (PHM) program for chronic disease management: comparison between implementation of intervention with and without a central population health coordinator (PHC) (non-randomly allocated).
**PROPS Study (Partnerships for Reducing Overweight and Obesity with Patient-centred Strategies) [PHM]**[2,3]	Primary care	An online weight management program integrated with PHM support. In weight-related PHM, nonclinical staff offer additional support by monitoring patient progress in an online program and conducting periodic outreach.<br>The study compared usual care (UC: general information about weight management via mail), online program alone (OP), and the combined intervention (CI: online program (OP) and weight-related PHM).
**Project Endo ECHO: Telementoring for care providers [PM]** [4–7]	Virtual clinics (for providers)	Project Endo ECHO (Extension for Community Healthcare Outcomes) is a telementoring program that builds new capacity to manage more patients in undeserved regions. This is done through shared knowledge networks with a multidisciplinary team of specialists, primary care providers (PCPs), and community health workers (CHWs). Endo ECHO (Endocrinology teleECHO Clinic) applies the ECHO model to complex diabetes. During teleECHO sessions, PCPs present real de-identified cases to receive guidance from specialists and peers on the network.
**The Reaction project (self-monitoring, registry) [PM]**[8]	Primary care	Diabetes patients self-monitor daily blood pressure and blood glucose, which were sent automatically to the clinician. Those that were deemed well controlled, after a two-week monitoring period, were asked to return the remote monitoring equipment (that were cycled to the next patient). The self-monitored data, the patient portal and the EPR can be used to create a comprehensive diabetes data management system.
**Prioritized telephone contacts [PM]**[9]	Community health centre	Prioritized telephone contacts by an attending and resident physician on top of usual care. The intervention patients were prioritized in the following order: (1) low-density lipoprotein (LDL) overdue, (2) HbA1c overdue, (3) last LDL > 99 mg/dL, (4) last HbA1c > 9%, and (5) last blood pressure > 130/80mm Hg.
**Diabetes Mellitus: Putting Evidence into Practice (DM-PEP) [PM]**[10,11]	Primary care	Individualized evidence-based guideline recommendations regarding metabolic testing and management (HbA1c, cholesterol, and blood pressure [BP]) based on weekly medical record review in clinical software PopMan. Recommendations were sent via e-mail to each intervention patient's primary care provider (PCP) by a nurse practitioner
**HEALTHeLINK (registry) [PHM]**[12]	Primary care	Electronic Health Record (HER) registry development across diverse systems and overcoming the challenges this presented. Change agents were deployed to move each group (primary care practices, EHR vendors, and diabetic patients) toward the goal of a communitywide diabetes registry and address the barriers that were encountered in the process. Practices used their registries for PHM by identifying and targeting patients in need of follow-up or specific diabetes-related care.
**Integrated disease management [PM]**[13]	Primary care	Implementation of combined population management, systems-based practice, and planned chronic illness care in primary care practices. Clinicians review a set of diabetes patient reports to identify gaps in quality of care. To address these gaps, a basic population care plan is created and executed. Finally, the impact of the tactics is reviewed.

**INtegrating DEPrEssioN and Diabetes treatmENT (INDEPENDENT) care [PHM]**[14,15]	Diabetes clinics and a hospital	A multi-component care model that enhances the skills of diabetes clinic teams to reduce depressive symptoms and improve diabetes risk factor management, patient-reported quality of life, and treatment satisfaction. It combines collaborative care, decision-support, and PHM. The intervention adds a non-physician care coordinator and consulting psychiatrist to the patient-diabetologist dyad to form a care team.
**Integrated diabetes care pilot [PHM]**[16]	Primary, secondary and community care (collaboration)	Collaborative working between primary, secondary and community care. The intervention included 1) clinical audit, 2) risk stratification, and 3) the multi-disciplinary virtual clinics in the community to discuss the outcomes of the clinical audits while taking into consideration the characteristics of the population. Also, to plan for improvement, proactively identify groups of patients at risk of complications from diabetes, and plan their care together.
**Mobile health (mHealth) Self-Management Intervention [PHM]**[17]	Primary care (federally qualified health centre [FQHC)]	Mobile health intervention to provide patient-centred information and promote engagement in self-management for patients who are at high risk for diabetic kidney disease (DKD). The intervention consisted of monthly telephone calls for 6 months by a (non-clinician) case manager and weekly, one-way informational text messages.
**Population-based evidence-based medicine [PM]**[18]	Pharmaceutical care	A population-based approach where clinical pharmacy specialists were responsible to apply evidence-based medicine for initiation, titration, and appropriate follow-up of ACE inhibitor therapy and in turn increase the proportion of patients with both cardiovascular disease (CAD) and diabetes who received target-dose ACE inhibitor therapy.
**Virtual pharmacy review (ViPRx) program [PHM]**[19]	Primary care	A virtual pharmacy review program with which pharmacists can remotely provide comprehensive pre-visit medication reviews for patients. The objectives were to positively affect clinical and cost-savings measures. Direct patient care services (face to face or by telephone) are not used in order to enhance pharmacist time spent.
**Preventing Heart Attacks and Strokes Everyday (PHASE) program [PHM]**[20,21]	Hospital care, regional clinic consortia, community health centres	An intervention to consistently deliver evidence-based preventive therapies for controlling blood glucose, low-density lipoprotein cholesterol (LDL-C), and blood pressure among patients with diabetes. This includes registry use. Levitz, 2021 describes a PHASE community implementation including grant funding, technical consultative support, formal learning community, and a structured quality improvement component.
**The Diabetes Master Clinician Program (DMCP) [PM]**[22]	Primary care	Tools to help primary care physicians and their patients attain HbA1c, LDL cholesterol, and blood pressure goals: an internet diabetes registry and group visits. The registry produces reports that facilitate population management of patients and make one-on-one office visits more efficient and of higher quality. Group visits empower patients to better self-manage their diabetes.
**International community health service PHM program (multilingual) [PHM]**[23]	Primary care	A multilingual multidisciplinary team that includes health educators, health-corps patient navigators, and community advocates with a collective fluency in more than a dozen languages and dialects. Members of the PHM team received training to provide self-management and prevention skills, using motivational interviewing techniques as part of the PHM intervention. A key effort of PHM interventions is follow-up with their primary care providers (PCPs).
**Registry population management [PM]**[24]	Hospital care	A redesigned workflow at the health care organization and practice levels, and a registry population management (RPM) application to support this new workflow. The RPM functions to accurately assess patients, determine their needs, provide interventions, and track the effectiveness of these interventions, all in real time, while freeing up staff time for important considerations like patient care
**Coordinated Population Health Approach [PM]**[25]	Primary care	An insurer-based diabetes educator (DE)–driven intervention that relied on systematic restructuring of primary care linking DE services through population health, practice redesign, and coordinated care. Two DEs were introduced as members of PC teams and worked to identify and refer DM patients considered at high risk

**Appendix Table 4 Population identification, risk stratification, and people centred actions in PHM interventions for type 2 diabetes**				
**PHM initiatives**	**1. Population identification**	**3. Risk stratification**	**4. People centred interventions**	
	**People with**		**(1) tailored interventions for predefined subpopulations**	**(2) interventions aimed to realize or improve the prerequisites for PM**
**Health information technology–enabled PHM program [PHM]**[38]	Diabetes, cardiovascular disease and hypertension, ≥18 years, at least 1 visit to a study practice within the prior 3 years.	No	No	Expansion of EHR tool and central population health coordinators (PHC)
**PROPS Study (Partnerships for Reducing Overweight and Obesity with Patient-centred Strategies) [PHM]**[39,40]	Hypertension or type 2 diabetes and BMI≥27 and <40kg/m2, aged 20-70, a recent primary care visit.	No	No	Working with a population health management team to improve outreach and incorporate PHM[A] support
**Project Endo ECHO: Telementoring for care providers [PM]** [32–34,41]	Complex diabetes (i.e. type 1 diabetes, type 2 diabetes on insulin, or type 2 diabetes with a HbA1c >9 %)[A], living in rural New Mexico, ≥18 years.	No	No	Sharing expertise with underserved regions
**The Reaction project (self-monitoring, registry) [PM]**[54]	Diabetes	Based on BP and BG[A]	Monitoring and appointment interval/ duration depends on self-monitored BP and BG[A]	Development of a diabetes data management system
**Prioritized telephone contacts [PM]**[42]	Diabetes	Based on LDL overdue, HbA1c overdue, LDL > 99 mg/dL, HbA1c > 9%, and BP > 130/80mm Hg[A]	Prioritization of (telephone) contacts based on stratification (3.)	No
**Diabetes Mellitus: Putting Evidence**	Diabetes with the highest HbA1c,[A] cholesterol levels, or most out-of-	No	Individualized evidence-based guideline recommendations	Clinical software PopMan

**into Practice (DM-PEP) [PM]**[43,44]	date results (determined with Clinical software PopMan)		(regarding testing, referral, and medication adjustment)	
**HEALTHeLINK (registry) [PHM]**[45]	Diabetes, ≥18 years.	Based on need for follow-up seeing lab testing, preventative services or diabetes-related care	Not described (focus on registry)	Yes, registry development, change agents (Clinical Transformation Partners), and mechanisms to sustain the population health collaborations are being explored
**Integrated disease management [PM]**[46]	Diabetes, at least 1 internal medicine visit over the past 18 months	Patients are color-coded based on a simple, clinical risk-stratification algorithm integrating three major evidence-based national treatment guidelines[B]	No	Yes, system redesign
**INtegrating DEPrEssioN and Diabetes treatmENT (INDEPENDENT) care [PHM]**[35,56]	Type 2 diabetes, depressive symptoms, 1 poorly-controlled cardiometabolic indicator (HbA1c, SBP, LDL) [A], ≥35 years old.	DS-EHR[A] system dashboard assists prioritizing participants based on most recent FBG, HbA1c, SBP, DBP, lipids, CVD history, PHQ-9[A] to a traffic light color scheme (green, adequate control; yellow, moderate control; red, poor control).[56] Care coordinators and consulting specialists reviewed data in the EHR[A] to stratify patients whose depression and/or cardiometabolic indices were poorly controlled.[35]	Prioritization in self-care support, proactive follow-up, outcome monitoring, evidence-based care prompts.[56] Individualized outreach, treatment intensification, and/or behavioural activation.[35]	Collaborative care (person-centred team care; population-based care; evidence-based care and measurement-based treatment to target) tailored to context and decision-support and electronic health record (DS-EHR) system and care coordinators are central figures
**Integrated diabetes care pilot [PHM]**[55]	Diabetes	Risk stratification of patients at risk of developing complications (HbA1c >9%, HbA1c <6.5% combined with insulin or sulphonylurea use, and eGFR <30 ml/min)[A] and other complex patients as requested by the primary healthcare professionals	Targeted interventions, use of data to determine changes in outcomes	Improved communication between primary care and specialists by virtual clinics

**Mobile health (mHealth) Self-Management Intervention [PHM]**[47]	Type 2 diabetes and poorly controlled hypertension, , aged 18-75, access to a mobile phone (for text messages and emails).	No	Tailored behavioural-educational components with a focus on disease self-management	STOP-DKD APP: an EHR[A] (identify the target population) and a second electronic platform that delivered evidence-based behavioural interventions
**Population-based evidence-based medicine [PM]**[48]	Diabetes and CAD (and no target-dose ACE inhibitor therapy) [A], >18 years.	Titration schedule based on: SBP,[A] potassium, creatinine clearance, and age	Lisinopril titration schedules: slow usual or accelerated	No
**Virtual pharmacy review (ViPRx) program [PHM]**[49]	Diabetes, ≥18 years.	No	The pharmacist outlines possible treatment options with a discussion of pros and cons before a scheduled visit	Yes, creation of the previsit virtual pharmacy review program ViPRx (virtual pharmacy review)
**Preventing Heart Attacks and Strokes Everyday (PHASE) program [PHM]**[37,50]	Diabetes	Trained diabetes care managers identify candidates for referral to health education classes based on an evidence-based treatment protocol	Referral to classes e.g. smoking cessation or diabetes education	Comprehensive registry, trained diabetes care managers.[50] Funding, technical assistance, supporting organizations to share best practices, quality improvement coaching, facilitating peer learning, infrastructure PHASE supporting sustainability.[37]
**The Diabetes Master Clinician Program (DMCP) [PM]**[51]	Diabetes	Practices are encouraged to invite their high-risk patients (not at goal for HbA1c, LDL, or BP)[A] to the group visits (identified via the diabetes registry)	For high-risk patients (at the greatest risk for complications): group visits and checks for time of last visit and plan a follow-up visit if needed	The diabetes registry, practice teams receive evidence-based training
**International community health service PHM program (multilingual) [PHM]**[52]	Diabetes and hypertension, no prescheduled PCP appointment or not seen a PCP >3 months[A]	No	Language	PHM[A] team received training

**Registry population management [PM]**[53]	Diabetes, ≥18 years.	The RPM[A] application helps determine cohorts of patients with specific criteria and needs	Determined cohorts are linked with actionable interventions, care is coordinated between the various providers	The RPM[A] application (intuitive user interface, "just-in-time" information)
**Coordinated Population Health Approach [PM]**[16]	High-risk diabetes (HbA1c>9%,[A] DM [A] -related emergency room visit, hospitalization, reported barriers to care)	DE determines patient needs and barriers after initial a face-to-face or telephonic diabetes self-management education and support (DSMES) session	Intervention ranged from chart review and plan of care recommendations to telephone-based or in-person individualized DSMES sessions depending on patient needs and preferences	Restructuring of primary care and employment of two diabetes educators to enhance care

[A] Abbreviations: ACE = Angiotensin-converting enzyme, BG = blood glucose, BP = blood pressure, , CAD = coronary artery disease DE = diabetes educator, DM = diabetes mellitus, DS-EHR = decision support and electronic health record, eGFR = glomerular filtration rate, EHR = electronic health record, FBG = fasting blood glucose, HbA1c = Hemoglobin A1C, LDL = low-density lipoprotein, PCP = primary care providers, PHM = Population Health Management, PHQ-9 = Patient Health Questionnaire-9, PHM = Population Health Management, RPM = registry population management, SBP = systolic blood pressure

[B] Joint National Committee VII, National Cholesterol Education Project Adult Treatment Panel III, and American Diabetes Association guidelines

Appendix Table 5 Detail on domains of the Triple Aim assessed for impact evaluation in PHM initiatives for T2DM (Table 3)			
**Initiative and origin**	**1. Population health**	**2. Quality of care**	**3. Direct and indirect costs**
**Health information technology–enabled PHM program [PHM]**[1]	Health outcomes: for diabetes LDL-C, HbA1c, and BP (For CVD: LDL-C. For HTN: BP)	Quality of care: increases in quality of care across all practices and for each chronic disease (goal attainment) Support: registry use for process metrics (e.g. obtaining tests) and outcome metrics (e.g. goal attainment)	No
**PROPS Study (Partnerships for Reducing Overweight and Obesity with Patient-centred Strategies) [PHM]**[2,3]	Health outcomes: BP, LDL, HDL, triglycerides, HbA1c, weight, BMI Behavioural/psychological factors: data on demographic factors Functioning/QoL: changes in patient-reported outcomes (e.g. weight-related quality of life, diet)	Effectivity: of online program with PHM or with usual care on weight change Responsiveness: use of and patient satisfaction with the interventions	No
**Project Endo ECHO: Telementoring for care providers [PM]** [4–7]	Health outcomes: self-reported health, disease-specific outcome measures (e.g. HbA1c), site visits, hospitalization outcomes, utilization (i.e. outpatient visits, hospitalizations, ED visits), provider behaviour regarding ADA guidelines (e.g. HbA1c testing, foot exams, and eye exams) Behavioural/psychological factors: self-reported demographic characteristics	Quality of care: guideline-based medication therapy prescriptions (e.g. metformin, antidepressant therapy), self-reported testing frequency (e.g. HbA1c testing, feet checks) Effectivity: cost-effectiveness of the intervention Responsiveness: self-reported health care quality Accessibility: changes in access to care in medically underserved communities (visits, travel time, delays)	Costs of care: Medicaid claims data on expenditures and general ledger cost accounting data Costs PM organization: time staff actually worked on the Endo ECHO project, resources the rural centres actually devoted to the project, and other shared unallocated indirect costs
**The Reaction project (self-monitoring, registry) [PM]**[8]	Health outcomes: BP, blood glucose	Responsiveness: patient' satisfaction with both the equipment and patient portal	No
**Prioritized telephone contacts [PM]**[9]	Health outcomes: HbA1c, LDL, BP Participation: number of phone calls, letters, visits avoided (overdue laboratory tests and/or the initiation of medication)	No	No

**Diabetes Mellitus: Putting Evidence into Practice (DM-PEP) [PM]**[10,11]	Health outcomes: diabetes-related metabolic risk factors (hyperglycaemia, hypertension, and hyperlipidaemia), urine microalbumin screening, and prescription of aspirin or ACE inhibitors	Quality of care: proportion of patients in each clinic's diabetes registry with risk factor testing (e.g. HbA1c, BP), last recorded values of each risk factor, and medication prescriptions<br>Responsiveness: percentage of specific recommendations on clinical testing and changes in therapy	No
**HEALTHeLINK (registry) [PHM]**[12]	Health outcomes: HbA1c, LDL, urine microalbumin/creatinine ratio, BP, documented influenza and pneumonia immunization	Quality of care: quarterly benchmark reports summarizing performance on key diabetes quality metrics (compared to community practice averages)<br>Support: registry use for reports on quality benchmarking	No
**Integrated disease management [PM]**[13]	Health outcomes: HgA1c, LDL, BP, pharmacologic management, comorbidities, smoking status	Quality of care: organizational performance (through planned care)<br>Accessibility: potential contribution of planned care to organizational improvement	No
**INtegrating DEPrEssioN and Diabetes treatmENT (INDEPENDENT) care [PHM]**[14,15]	Health outcomes: depression and CVD risk factors (e.g. HbA1c, BP), self-reported medical / health use history<br>Behavioural/psychological factors: sociodemographic characteristics, self-care activities, depressive symptoms<br>Functioning/QoL: health related QoL	Responsiveness: patient reported treatment satisfaction	Costs of care: self-reported direct non-medical costs, health expenditures, within-trial cost-utility<br>Productivity losses: self-reported indirect costs (lost productivity associated with illness or premature mortality)
**Integrated diabetes care pilot [PHM]**[16]	No	Quality of care: changes in self-reported knowledge of diabetes management and referral system<br>Effectivity: change in the managing diabetes (virtual clinics); change in ways of working between GPs, primary care nurses and diabetes specialists	No
**Mobile health (mHealth) Self-Management Intervention [PHM]**[17]	Health outcomes: SBP<br>Participation: engagement (number of phone calls), and whether greater participation in the intervention was associated with a greater impact	No	No

**Population-based evidence-based medicine [PM]**[18]	Health outcomes: creatinine, potassium, and BP	Patient safety: safety and tolerability of each titration schedule, reasons the target dose was not achieved	No
**Virtual pharmacy review (ViPRx) program [PHM]**[19]	Health outcomes: medication use and adherence, clinical outcomes (HbA1c, LDL, diabetic nephropathy), medical, surgical, and immunization history	Effectivity: time taken and ability to achieve the desired outcomes with pre-visit reviews	Costs of care: prescription drug costs
**Preventing Heart Attacks and Strokes Everyday (PHASE) program [PHM]**[20,21]	Health outcomes: changes in glucose, lipid, risk factor control, BP control, rates of prescription of ACE/ARBs and statins [21]	Support: registry to measure performance on important dimensions of care and service	No
**The Diabetes Master Clinician Program (DMCP) [PM]**[22]	Health outcomes: HbA1c, LDL, or BP (ADA goals)	Responsiveness: patient report cards on clinical measures, patient satisfaction	Costs of care: cost savings per year per patient
**International community health service PHM program (multilingual) [PHM]**[23]	Health outcomes: for diabetes HbA1c (for hypertension - BP)	Quality of care: quality improvement through PHM (treatment guidelines and goals met)	No
**Registry population management [PM]**[24]	No	Effectivity: patient review rate of nurse and time between patient identification and mailing reminder letter	Costs of PM organization: effect of pay-for-performance on adaptation of the RPM application
**Coordinated Population Health Approach [PM]**[25]	Health outcomes: HbA1c, LDL, BMI	No	No
Abbreviations: ACE = angiotensin-converting enzyme, ACC = ambulatory care coordinators, ARB = angiotensin-receptor blocker, BMI = Body Max Index, BP = blood pressure, CHW = community health worker, CVD = cardiovascular disease, ED = emergency department, FBG = fasting blood glucose, HbA1c = haemoglobin A1C, HDL = high-density lipoprotein, HTN = hypertension, LDL = low-density lipoprotein, PCP = primary care provider, PHM = Population Health Management, PHQ-9 = Patient health questionnaire 9, QoL = quality of life, RPM = Registry Population Management, SBP = systolic blood pressure			

Appendix Table 6 PHM initiatives for type 2 diabetes: quality improvement processes	
**PHM initiatives with quality improvement processes**	**6. Quality improvement process**
**Health information technology–enabled PHM program [PHM]**[1]	Process mapping and Plan-Do-Study-Act (PDSA) cycles
**PROPS Study (Partnerships for Reducing Overweight and Obesity with Patient-centred Strategies) [PHM]**[2,3]	Incorporation of input from patients, PCPs (primary care providers), and other stakeholders
**HEALTHeLINK (registry) [PHM]**[12]	PDSA cycles and feedback on the quality of practices' data and the benchmark reports
**Integrated disease management [PM]**[13]	Plan-Do-Check-Act (PDCA) cycles, phased program development (initial design, formal pilot, and the broad program dissemination), practice customization of the intervention, and cross-organizational learning and monitoring of implementation process
**Integrated diabetes care pilot [PHM]**[16]	Clinical audits to systematically care (which creates a learning culture to deliver change), and co-design based on principles of service improvement and continual improvement process
**Population-based evidence-based medicine [PM]**[18]	Quality improvement activities based on clinical, population, management and patient tracking data
**Virtual pharmacy review (ViPRx) program [PHM]**[19]	Provider feedback and stepwise program implementation to ensure that any early concerns that arose could be quickly addressed
**Preventing Heart Attacks and Strokes Everyday (PHASE) program [PHM]**[20,21]	KPNC PHASE quality improvement program with regular reports on hypertension prevalence and LDL-C and HbA1c control, regular updates of the evidence-based cardiovascular risk factor control algorithm for step therapy, and individual quality improvement coaching (e.g. regarding data quality)
**International community health service PHM program (multilingual) [PHM]**[23]	User feedback to improve the preparation and training for staff involved with PHM
**Registry population management [PM]**[24]	Just-in-time knowledge access tool, user suggestions on design, and PDSA cycle

Appendix Table 7 Data warehouse availability and use in PHM initiatives for type 2 diabetes	
**PHM initiatives with a data warehouse**	**Data warehouse availability and use**
**Health information technology–enabled PHM program [PHM]**[1]	Health IT clinical registry, used for population identification
**PROPS Study (Partnerships for Reducing Overweight and Obesity with Patient-centred Strategies) [PHM]**[2,3]	EHR (Electronic Health Record), used for population identification
**Prioritized telephone contacts [PM]**[9]	Diabetes registry (on clinical measures), used for population identification
**Diabetes Mellitus: Putting Evidence into Practice (DM-PEP) [PM]**[10,11]	EMR (Electronical Medical Record), used for population identification
**INtegrating DEPrEssioN and Diabetes treatmENT (INDEPENDENT) care [PHM]**[14,15]	Medical records (e.g. EHR for decision support), used for population identification
**Mobile health (mHealth) Self-Management Intervention [PHM]**[17]	EHR, used for population identification
**Population-based evidence-based medicine [PM]**[18]	Web-based tracking database (HealthTRAC,) containing membership, administrative and clinical data, used for population identification
**Virtual pharmacy review (ViPRx) program [PHM]**[19]	EMR's health registries database, payer data, and registry of diabetes patients (diabetes health registry list), used for population identification
**Preventing Heart Attacks and Strokes Everyday (PHASE) program [PHM]**[20,21]	Diabetes registry with clinical data (outpatient diagnostic codes, pharmacy data, hospitalization records), used for population identification
**International community health service PHM program (multilingual) [PHM]**[23]	PHM reports (generated from the EHR), used for population identification
**Registry population management [PM]**[24]	RPM (registry population management) application combining clinical, claims, laboratory and administrative data, used for population identification

References

1. Ashburner JM, Horn DM, O'Keefe SM, Zai AH, Chang Y, Wagle NW, et al. Chronic Disease Outcomes From Primary Care Population Health Program Implementation. Am J Manag Care. 2017;23(12)(December):728–35.

2. Baer HJ, De La Cruz BA, Rozenblum R, Nolido N V., Orav EJ, Metzler K, et al. Integrating an online weight management program with population health management in primary care: Design, methods, and baseline data from the PROPS randomized controlled trial (Partnerships for Reducing Overweight and Obesity with Patient-centered Strate. Contemp Clin Trials [Internet]. 2020;95(May):1–9. Available from: https://doi.org/10.1016/j.cct.2020.106026

3. Baer HJ, Rozenblum R, De La Cruz BA, Orav EJ, Wien M, Nolido N V., et al. Effect of an Online Weight Management Program Integrated with Population Health Management on Weight Change: A Randomized Clinical Trial. JAMA - J Am Med Assoc. 2020;324(17):1737–46.

4. Bouchonville MF, Paul MM, Billings J, Kirk JB, Arora S. Taking Telemedicine to the Next Level in Diabetes Population Management: a Review of the Endo ECHO Model. Curr Diab Rep [Internet]. 2016;16(96):1–7. Available from: http://dx.doi.org/10.1007/s11892-016-0784-9

5. Bouchonville MF, Hager BW, Kirk JB, Qualls CR, Arora S. Endo echo improves primary care provider and community health worker self-efficacy in complex diabetes management in medically underserved communities. Endocr Pract. 2018;24(1):40–6.

6. Blecker S, Lemieux E, Paul MM, Berry CA, Bouchonville MF, Arora S, et al. Impact of a primary care provider Tele-mentoring and community health worker intervention on utilization in Medicaid patients with diabetes. Endocr Pract. 2020;26(10):1070–6.

7. Paul MM, Saad AD, Billings J, Blecker S, Bouchonville MF, Chavez C, et al. A telementoring intervention leads to improvements in self-reported measures of health care access and quality among patients with complex diabetes. J Health Care Poor Underserved. 2020;31(3):1124–33.

8. Clarke M, Fursse J, Gokalp H, Sharma U, Jones RW. Whole population management of patients with diabetes. IEEE EMBS Spec Top Conf Healthc Innov Point-of-Care Technol. 2014;197–8.

9. Fischer HH, Villacres A, Durfee MJ, McCullen K, MacKenzie TD. Diabetes population management by telephone visits. Telemed e-Health. 2011;17(5):396–8.

10. Grant RW, Hamrick HE, Sullivan CM, Dubey AK, Chueh HC, Cagliero E, et al. Impact of population management with direct physician feedback on care of patients with type 2 diabetes. Diabetes Care. 2003;26(8):2275–80.

11. Grant RW, Cagliero E, Sullivan CM, Dubey AK, Estey GA, Weil EM, et al. A Controlled Trial of Population Management. Diabetes Mellitus: Putting Evidence into Practice (DM-PEP). Diabetes Care. 2004;27(10):2299–305.

12. Heider AR, Maloney N, Satchidanand N, Allen G, Mueller R, Gangloff S, et al. Developing a Community-Wide Electronic Health Record Disease Registry in Primary Care Practices: Lessons Learned from the Western New York Beacon Community. Vol. 2, eGEMs (Generating Evidence & Methods to improve patient outcomes). 2014. p. Art.7 p.1-8.

13. Kimura J, DaSilva K, Marshall R. Population management, systems-based practice, and planned chronic illness care: Integrating disease management competencies into primary care to improve composite diabetes quality measures. 2008. p. 11;13-22.

14. Kowalski A, Poongothai S, Chwastiak L, Hutcheson M, Tandon N, Khadgawat R, et al. The INtegrating DEPrEssioN and Diabetes treatmENT (INDEPENDENT) study: design and methods to address mental healthcare gaps in India. Physiol Behav. 2017;September(60):113–24.

15. Ali MK, Chwastiak L, Poongothai S, Emmert-Fees KMF, Patel SA, Anjana RM, et al. Effect of a Collaborative Care Model on Depressive Symptoms and Glycated Hemoglobin, Blood Pressure, and Serum Cholesterol among Patients with Depression and Diabetes in India: The INDEPENDENT Randomized Clinical Trial. JAMA - J Am Med Assoc. 2020;324(7):651–63.

16. Kozlowska O, Attwood S, Lumb A, Tan GD, Rea R. Population health management in diabetes care: Combining clinical audit, risk stratification, and multidisciplinary virtual clinics in a community setting to improve diabetes care in a geographically defined population. An integrated diabetes care pilot i. Int J Integr Care. 2020;20(4):1–11.

17. Lewinski AA, Patel UD, Diamantidis CJ, Oakes M, Baloch K, Crowley MJ, et al. Addressing diabetes and poorly controlled hypertension: Pragmatic mHealth self-management intervention. J Med Internet Res. 2019;21(4):1–13.

18. Mcconnell KJ, Zadvorny EB, Denham AM, Kasten SL, Hutka KA, Koetting CR, et al. Angiotensin-converting Enzyme Inhibitor Therapy for Coronary Artery Disease and Diabetes Mellitus. Am J Manag Care. 2007;13(10):560–6.

19. Muraywid B, Butkievich LE, Myers B. Effect of a virtual pharmacy review program: A population health case study. J Manag Care Spec Pharm. 2020;26(1):24–9.

20. Rana JS, Karter AJ, Liu JY, Moffet HH, Jaffe MG. Improved Cardiovascular Risk Factors Control Associated with a Large-Scale Population Management Program Among Diabetes Patients. Am J Med [Internet]. 2018;131(6):661–8. Available from: https://doi.org/10.1016/j.amjmed.2018.01.024

21. Levitz C, Jones M, Nudelman J, Cox M, Camacho D, Wielunski A, et al. Reducing cardiovascular risk in patients with diabetes: An evidence-based, population health management program. J Healthc Qual. 2021;00(00):1–10.

22. Shahady E. The Florida Diabetes Master Clinician Program: Facilitating increased quality and significant cost savings for diabetic patients. Clin Diabetes. 2008;26(1):29–33.

23. Tsui EW, Wang G, Zahler A, Simoyan OM, White M V., Mckee M. A multilingual population health management program. J Ambul Care Manage. 2013;36(2):140–6.

24. Zai AH, Grant RW, Estey G, Lester WT, Andrews CT, Yee R, et al. Lessons from Implementing a Combined Workflow-Informatics System for Diabetes Management. J Am Med Informatics Assoc. 2008;15(4):524–33.

25. Zupa MF, Arena VC, Johnson PA, Thearle MB, Siminerio LM. A Coordinated Population Health Approach to Diabetes Education in Primary Care. Diabetes Educ. 2019;45(6):580–5.
